# Treatment strategies and prognostic insights for lacrimal gland adenoid cystic carcinoma: a review

**DOI:** 10.1007/s12672-025-02468-5

**Published:** 2025-05-22

**Authors:** Xinyun Wang, Huiling Ma, Ying Chen, Menghui Zhang, Sisi Liu, Huiyan Li, Xiawei Wang, Hongguang Cui

**Affiliations:** 1 Department of Ophthalmology, The Fourth Affiliated Hospital of Zhejiang University School of Medicine, Yiwu, China; 2 Department of Ophthalmology, The First Affiliated Hospital of Zhejiang University School of Medicine, Hangzhou, China

**Keywords:** Adenoid cystic carcinoma, Lacrimal gland, Surgery, Chemotherapy, Radiotherapy, Molecular targeted therapy

## Abstract

Adenoid cystic carcinoma (ACC) is the most common type of malignant tumor in lacrimal gland cancer. The primary treatment approach involves eye-preserving surgery combined with adjuvant radiotherapy, which has proven effective in maintaining visual function and achieving favorable local control with minimal toxicity. However, the 5-year survival rate for ACC of the lacrimal gland remains below 60%. Recently, novel adjuvant therapies, including neoadjuvant intra-arterial chemotherapy, proton radiotherapy, and neutron radiotherapy, have significantly improved survival outcomes. Despite these advances, the rarity of lacrimal gland adenoid cystic carcinoma (LGACC) limits comprehensive studies on long-term survival and the potential for late toxicity, underscoring the need for further research. Additionally, recent findings on pathogenic mechanisms and proteomic abnormalities in LGACC offer a foundation for developing targeted therapies, paving the way for more personalized treatments. This article reviews contemporary treatment strategies and prognostic insights for LGACC, focusing on recent advancements and their implications for patient survival.

## Introduction

Adenoid cystic carcinoma (ACC) is an uncommon malignant neoplasm of the secretory adenocarcinoma type, most commonly found in the salivary glands. Lacrimal gland adenoid cystic carcinoma (LGACC) is even rarer, with only over 100 cases reported in the literature to date [1, 2]. LGACC is the most prevalent malignant epithelial tumor of the lacrimal gland, accounting for approximately 1.6% of all orbital tumors [3–8]. Common clinical manifestations include proptosis, eyelid swelling, lacrimal masses, and ptosis. Pain, along with bone destruction and nerve invasion, is a distinguishing feature of LGACC. Imaging studies, such as magnetic resonance imaging (MRI) and computed tomography (CT), typically reveal solid tumors, sometimes with bone erosion, local invasion, or lymphatic metastasis. Diagnosis of LGACC is primarily based on histopathological evaluation.

The median age of onset for LGACC ranges between 38.9 and 41 years [1–3]. Females are generally more affected than males, with women comprising 58%–70% of reported cases [1, 3, 4]. However, a higher proportion of male patients has been observed in some studies [10], likely due to the rarity of the disease, which makes it challenging to establish consistent epidemiological trends.

Treatment approaches for LGACC vary considerably across institutions, and no standardized protocol has been universally established for different stages and types of this carcinoma [3, 5–13]. Historically, orbital exenteration was the primary surgical intervention, often followed by postoperative radiotherapy. In recent years, additional adjuvant therapies, such as neoadjuvant intra-arterial chemotherapy (NIAC), proton radiotherapy, stereotactic radiosurgery, neutron radiotherapy, and carbon-ion radiotherapy [8, 10, 12, 14–17], have been introduced.

Some of the above therapies can even achieve local control (LC) in appropriately selected cases where the tumor extensively involves orbital soft tissues [8, 18]. The 5-year survival rate for LGACC has been below 60% over the past few decades [7, 13, 25, 26], and the introduction of NIAC in 2006 improved the 5-year survival rate to 83.3% [27].

LGACC is characterized by a high risk of late recurrence, bone destruction, and distant metastasis (DM) [19, 20]. Recurrence rates are associated with factors such as primary tumor site, tumor size, age, and lymph node involvement [14]. Perineural infiltration (PNI) is also a risk factor for local recurrence [22]. Additionally, PNI also provide a pathway for tumor cell spread without vascular or lymphatic infiltration [23, 24]. In distant metastasis cases, the most common sites are the lungs, followed by the bones and liver. A recent study by Joshua et al. [22] found that 25% of patients experienced local recurrence, and 34% developed distant metastasis.

The initial reported 5-year survival rate of LGACC was below 60% [1, 9, 15, 16]. Hence, following the introduction of NIAC by Tse et al. [17] in 2006, promising outcomes were observed, with a 5-year survival rate reaching 83.3% and the cumulative 5-year recurrence rate in NIAC treated group was decreased to 23.8%.

The prognosis of LGACC is influenced by several factors, including early diagnosis, histological grade, treatment strategy, patient age, sex, tumor type, and stage. Among these, tumor stage and histological subtype are critical determinants of outcomes. Studies suggested that eye-sparing surgery with adjunctive radiotherapy may achieve optimal disease control in tumors staged T1 or T2, but not in those staged T3 or T4 [18]. Additionally, patients with a predominantly basaloid subtype tend to have poorer outcomes than those with a chiefly cribriform subtype [19, 20], highlighting the need for future research with histopathology exploring the biological behavior of neoplastic cells.

Due to the rarity and aggressive nature of LGACC, establishing universally accepted treatment guidelines and clinical research remains a challenge. To improve understanding of this carcinoma, this paper reviews and synthesizes global literature up to 2024, aiming to consolidate current treatment options and prognostic factors for LGACC and explore a more unified treatment approach.

## Methods

**Fig. 1:**
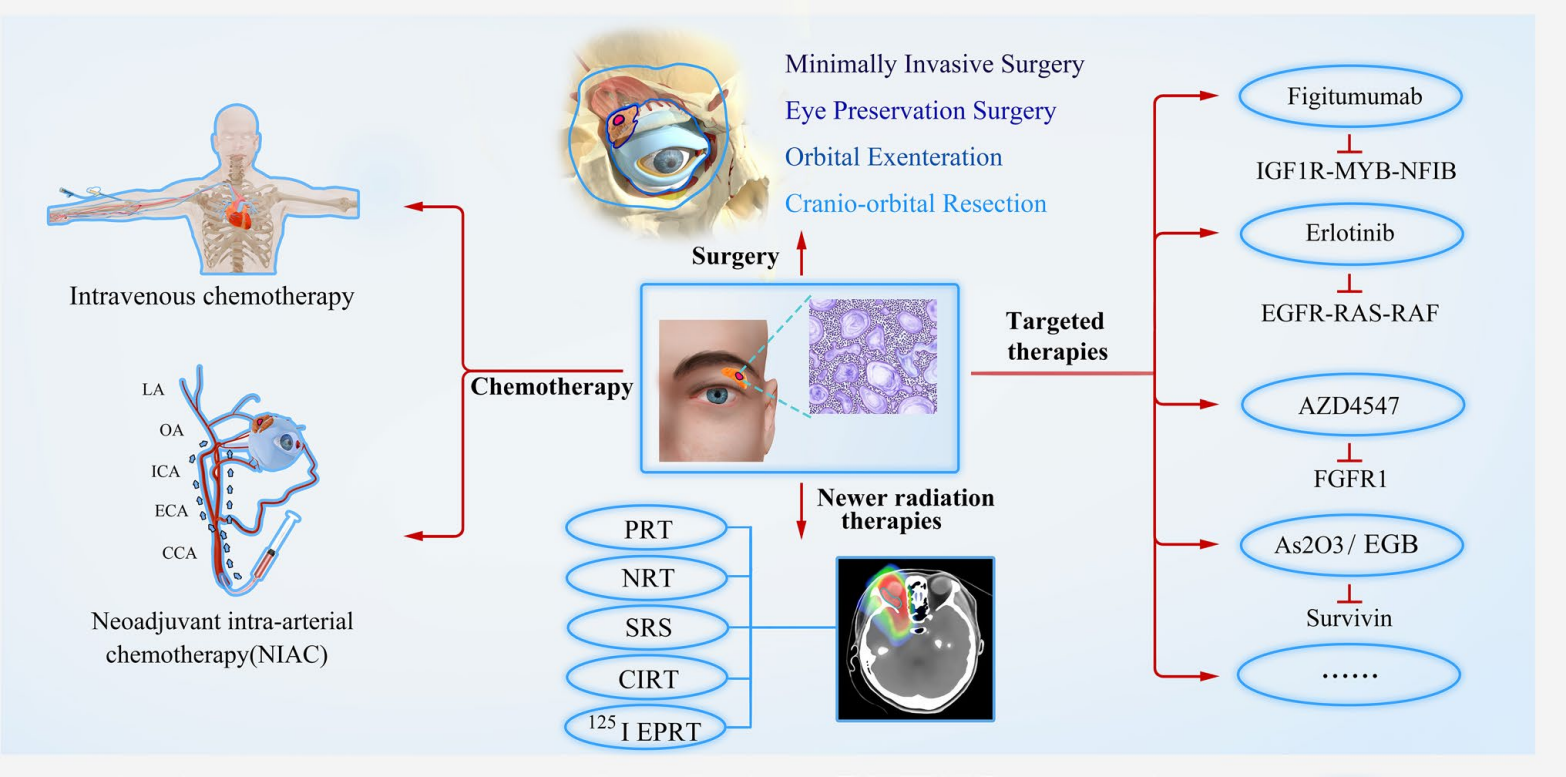
Summary of Current Treatment Methods of LGACC. Abbreviations: LA, Lacrimal artery; OA, Ophthalmic artery; ICA, Internal carotid artery; ECA, External carotid artery; CCA, Common carotid artery; PRT, Proton radiotherapy; NRT, Neutron radiotherapy; SRS, Stereotactic radiosurgery; CIRT, Carbon-ion radiation therapy; 125I EPRT, 125I plaque radiotherapy; IGF1R, Insulin Like Growth Factor 1 Receptor; NFIB, Nuclear Factor I B; EGFR, Epidermal Growth Factor Receptor; RAS, Rat sarcoma; FGFR1, Fibroblast Growth Factor Receptor 1; As2O3, Arsenic trioxide; EGB, Extract of Ginkgo biloba

We conducted a literature search on the PubMed and Web of Science databases for all published English-language articles on LGACC from 1967 to 2024. The search strategy included the following terms: (Adenoid cystic carcinoma [Title/Abstract]) AND (lacrimal gland [Title/Abstract]) and yielded a total of 269 articles. Additional studies relevant to recent advancements and findings in LGACC treatment were also included in this review (Fig. 1).

## Imaging

Imaging is essential to determine tumor size, location, extent, and characteristics.

MRI provides superior soft tissue resolution, revealing tumor relationships with surrounding tissues. In LGACC, MRI often shows medium-to-low signal intensity on T1-weighted images (T1WI) and medium-to-high signal intensity on T2-weighted images (T2WI), with contrast enhancement highlighting areas of cystic changes [32]. CT images of typical LAGCC demonstrate irregularly shaped, densely hyperattenuating masses within the lacrimal gland. These lesions typically exhibit moderate to marked enhancement following contrast administration, with areas of non-enhancing cystic or necrotic components [21].In addition, previous study by Williams et al. [10] reported radiographic evidence of bone involvement in 87.5% of patients. In this situation, CT is an indispensable tool for identifying skeletal involvement, which plays a crucial role in determining the appropriate surgical approach.

## Surgical treatments

The primary goal objective in managing LGACC is to achieve effective LC while minimizing the risk of distant metastasis and recurrence. Surgery remains the cornerstone of treatment and is often supplemented by radiotherapy and chemotherapy. Surgical approaches are largely determined by tumor stage and imaging findings, as classified by the American Joint Committee on Cancer (AJCC).

Surgical management of LGACC aims for complete tumor resection while preserving the patient’s quality of life. However, no standardized surgical approach has been universally accepted. Surgical options vary based on tumor stage and imaging characteristics, as classified by the AJCC. The key surgical approaches include:

### Surgical approaches

#### Eye preservation surgery

For tumors that have not extensively invaded ocular structures, eye-preserving surgery aims to remove the tumor while maintaining visual function. Eye-preserving surgery, combined with adjuvant radiotherapy, is the most common strategy.

#### Orbital exenteration

A more radical procedure involving removal of the entire orbital contents, including the eye, eyelids, and surrounding tissues. This approach is generally reserved for larger or more advanced tumors where conservative surgery may not ensure complete tumor clearance.

#### Minimally invasive surgery

Appropriate for early-stage tumors (T1 or T2), this approach minimizes impact on surrounding healthy tissue and reduces postoperative morbidity.

#### Cranioorbital resection

For tumors that extend beyond the orbit, cranioorbital resection may be necessary, involving the removal of both orbital and cranial structures to address invasion into adjacent cranial areas.

### Surgical challenges

Common surgical challenges in LGACC include determining the scope of resection preoperatively and achieving negative margins to prevent local recurrence and metastasis.

Early studies, such as Wright et al. [2] indicated that disease-free survival rates were not significantly different based on whether cranioorbital resection was performed. Similarly, a study from MD Anderson Cancer Center found that radical surgeries like orbital exenteration with bone removal did not improve recurrence, metastasis, or mortality rates but instead negatively impacted patients' quality of life due to functional and aesthetic impairments [5]. Therefore, it is crucial to determine the most suitable surgical approach prior to the procedure.

Preoperative imaging can provide clues about the size of the tumor. Tumor size is an important criterion that guides doctors in determining the most suitable surgical method. Patients with early-stage T1 or T2 tumors may be candidates for minimally invasive surgery. Patients with tumors < T3 are recommend to eye-preserving surgery with radiotherapy. Patients with tumors > T3 (classified by the AJCC’s sixth edition as 2.5–5 cm) may require more aggressive approaches like orbital exenteration, who were reported by Ahmad et al. [5] might experience higher rates of local recurrence, lymph node involvement, distant metastasis, and poorer overall survival (OS). Imaging can also determine whether there is bone invasion, which we have concluded in imaging paragraph. In cases of bone invasion in the lacrimal fossa, a more extensive surgical approach is recommended to remove the affected bone wall.

New technologies, such as the electrical impedance tumor detection system (ITDS), have shown promise in ensuring negative margins during surgery. A study by Fattahi et al. [22] demonstrated that ITDS could accurately detect tumor and margin boundaries consistent with histopathological results. Even if the surgical margin is negative, postoperative follow-up is also very important. According to existing studies, the average time for recurrence in LGACC patients is 66 months, so lifelong follow-up is required after surgery, especially close follow-up within the first 5 years, combined with orbital MRI or orbital CT.

In conclusion, while surgical treatment of LGACC remains complex and lacks standardization, advancements in AJCC staging, imaging modalities, and innovative technologies like ITDS continue to improve surgical accuracy and outcomes.

## Chemotherapy

Most traditional chemotherapy studies focus on head and neck ACC (HNACC), where intravenous chemotherapy alone has shown limited effectiveness. Among the available options, only platinum and anthracyclines exhibit notable anti-tumor activity, although their use is limited by toxicity [23], including myelosuppression, septicemia, renal insufficiency. Because cisplatin and doxorubicin have relatively strong relatively anti-tumor effect and low biological toxicity, they are also widely used in the treatment of HNACC. Given the similarities in embryonic origin and biological behavior between LGACC and HNACC, cisplatin and doxorubicin have also been commonly used for LGACC [35, 36]. In LGACC, cisplatin and doxorubicin are frequently employed due to LGACC’s similar embryogenesis and biological behavior as HNACC [24, 25]. However, a more effective and less toxic option known as NIAC has emerged.

NIAC, initially studied in 1998 [87], delivers high concentrations of chemotherapy drugs directly to the tumor site, promoting tumor cell death prior to surgery, Several studies have also proven the anti-tumor effect with decreased tumor volume and decreased 5-year disease-specific mortality (16.7%) after NIAC chemotherapy compared with the conventional group (57.1%) [27]. Several studies have shown that the tumor volume decreases after NIAC chemotherapy, and the prognosis of patients is good [11]. In a 2013 follow-up study of NIAC treatment, the 10-year disease-free survival rate of patients with preserved lacrimal artery functionality reached 100% [8]. NIAC has been proven to inhibit the growth of tumor cells. Immunohistochemistry (IHC) of the excised specimen in a patient with LGACC (T4cN0M0 based on AJCC 8th edition) after NIAC treatment showed positive apoptotic markers and lower tumor burden compared to the incisional biopsy specimen [26]. These results suggest that NIAC is indeed effective in killing tumors and is effective in improving local disease control and overall disease-free survival.

Despite its benefits, NIAC poses potential risks, such as temporary facial nerve paralysis, facial swelling, vascular events leading to vision loss, anterior ischemia, necrosis of the ipsilateral eyelid and eyeball, forehead skin pigmentation, neutropenia, and thrombocytopenia [8, 27]. Additionally, another case of a patient treated with NIAC who subsequently developed treatment-related acute myeloid leukemia (AML) highlights the need for careful consideration of the risks and benefits associated with NIAC treatment [8].

Selecting the appropriate blood vessel for NIAC is essential to maximize drug delivery and minimize systemic toxicity. The lacrimal artery, typically supplied by branches from both the internal (ICA) and external (ECA) carotid arteries, can serve as a route for targeted drug administration. The therapeutic effect of NIAC via ICA or ECA varies. In a previous study, patients who received NIAC through the ECA showed no significant histopathological damage to key ocular structures or compromised visual function, whereas chemotherapy through the ICA may result in visually significant thrombotic vascular events [28]. In addition, studies comparing the ICA and ECA pathways in animal models have found that the ICA pathway produces greater ocular toxicity [29]. Moreover, smaller catheters for direct ocular artery insertion [30] have improved the precision of chemotherapy delivery.

In conclusion, NIAC has emerged as an effective treatment for LGACC, enhancing both survival and disease control.

## Newer radiation therapies

Postoperative adjuvant radiotherapy is integral to managing LGACC, primarily aiming to control local disease [2, 4, 15]. External beam photon radiation therapy is conventionally used, though its effectiveness is limited. This has driven research into more advanced radiotherapy methods for improved outcomes.

### Proton radiotherapy (PRT)

Proton radiation therapy has been applied to treat intraocular tumors such as choroidal melanoma [31] and head and neck cancers [32]. In LGACC, proton therapy is particularly suitable for patients with locally advanced tumors or residual lesions after surgery. Proton therapy is able to focus high doses on the tumor area, reducing damage to surrounding normal tissue. In a study [33] on seven patients with LGACC who received proton therapy following eye preservation surgery, a median follow-up duration of 27.1 months showed no signs of local recurrence. Another cohort of 18 patients with LGACC had a median follow-up period was 12.9 years, with three patients succumbing to metastatic disease within 4.2 years post-treatment and four cases of local recurrence. Despite these challenges, the OS and vision quality were promising, with most patients retaining 20/40 or better vision for an average of 3 years post-treatment [34].

While proton radiotherapy demonstrates superior therapeutic efficacy, studies have identifiedside effects. Advanced assessments, including optical coherence tomography,is used to detect subclinical injuries, such as optic nerve abnormalities [46].Irradiating the orbit and periorbital region always carries considerable risks of toxicity. A study [33] showed that reaching a maximum corneal dose of > 36 Gy relative biological effectiveness (RBE) can increase the risk of chronic grade ≥ 3 ocular toxicity, though eye-deviation techniques may help keep doses within safer limits.

### Neutron radiotherapy (NRT)

Neutron radiotherapy has been explored as a supplementary treatment for LGACC to enhance therapeutic outcomes. A study of 11 patients treated with neutron therapy following surgery found a 5-year local control rate (LR) of 80%, yet late recurrence and distant metastasis remained challenges [35]. Following a median 75-month follow-up, six patients had died, with three local recurrences and four cases of distant metastasis reported, highlighting the importance of continuous monitoring.

### Stereotactic radiosurgery (SRS)

SRS delivers a high-dose radiation beam to an intracranial target to cause lesions or remove pre-existing lesions. This technique has evolved to treat diverse tumor shapes effectively, which allows for non-open surgical treatment of intracranial lesions, thereby minimizing surgical trauma. Several research suggest that SRS should become the preferred treatment for patients with brain metastases with unresectable tumors [36, 37]. In one case, a patient with LGACC and meningeal metastasis received multiple rounds of SRS and showed no signs of disease and had a good quality of life after 27 months. While effective, SRS still has limitations such as high costs, specialization requirement, and side effects including fatigue, headaches, brain swelling, nausea and vomiting [38].

### Carbon-ion radiation therapy (CIRT)

CIRT offers high linear energy transfer rate and RBE and better dose-targeting properties than photon radiotherapy [39], making it a promising option for radioresistant tumors. In a cohort of 33 patients with invasive lacrimal gland carcinoma (LGC) beyond the orbit, CIRT achieved a 5-year LR of 62% and OR of 65% after a median follow-up of 53.7 months, although optic nerve disorders, cataract glaucoma, retinopathy, and central nervous system necrosis were reported as side effects [40]. A study by Akbaba et al. [41] included 24 patients with malignant LGC who received CIRT and reported a 2-year LC, OS, and disease-free survival of 93%, 96%, and 87%, respectively, with CIRT showing lower toxicity than other radiotherapy modalities.

### ^125^I episcleral plaque radiotherapy (^125^I EPRT)

^125^I episcleral plaque radiotherapy is commonly used for certain intraocular malignancies, but application for LGACC is limited. Shields et al. [10] reported four cases where iodine-125 plaques (target dose: 50 Gy) were placed post-surgery with no tumor recurrence during follow-up. Lewis et al. [42] also reported no local tumor recurrence after a 4-year follow-up, and Zhang et al. [43] confirmed the killing effect of radioactive ^125^I particles in targeting LGACC tissue.

While various advanced radiotherapy techniques show potential for LGACC, follow-up is essential to evaluate outcomes such as late recurrence, metastasis rates, survival, and ocular toxicity.

## Molecular mechanism and potential targeted therapy for LGACC

Molecular genetic studies of LGACC have revealed specific gene mutations and gene fusion that could serve as targets for future tumor targeted therapies. Given LGACC’s resistance to existing treatments, researchers are exploring potential biological targets for developing targeted therapies.

Persson et al. [44] identified the MYB-NFIB gene fusion, resulting from a translocation between chromosomes 6 and 9 [t (6, 9) (q22-23, p23-24)], in various ACCs, including LGACCs. The mechanism is either through copy number gain or enhancer hijacking [45, 46]. This is a pro-oncogenic phenomenon whereby the upregulation of an oncogene is driven by enhancer repositioning leading to altered chromatin interactions and ultimately increased oncogene expression [47, 48]. Andersson et al. [49] found that this gene fusion in ACC promotes tumor growth by activating the Akt-dependent insulin-like growth factor 1 receptor (IGF1R) pathway, which regulates the cell cycle, RNA modification, and DNA replication. Morelli et al. [50] reported a phase I drug clinical trial of figitumumab, an IGF1R monoclonal antibody, combined with PF00299804, a pan–human epidermal growth factor receptor (EGFR) inhibitor, stabilized disease progression for 6 months. Calvo et al. [51] reported that Figitumumab and dacomitinib stabilized the disease in a single patient with ACC for and 1.5 years. These results suggested that the IGF1R-MYB-NFIB axis may be a promising therapeutic target for ACC. However, there are no clinical trials for MYB-NFIB, and we will continue to focus on its use in the treatment of LGACC in the future.

In addition, a therapy targeting the NOTCH gene mutation and the EGFR-RAS-RAF cascade of the EGFR pathway has received widespread attention [52]. Nie et al. [53] reported the case of a patient with metastatic LGACC treated with Erlotinib, an EGFR tyrosine kinase inhibitor (EGFR-TKI). After 1 month, the lung tumor showed significant shrinkage, and the treatment remained effective for 14 months. This outcome suggests that EGFR-TKI therapy may offer a viable approach for managing metastatic LGACC with tolerable side effects.

Doddapaneni et al. [54] found that fibroblast growth factor receptor 1 (FGFR1) signaling was significantly upregulated following NIAC in LGACC. When LGACC cells were treated with the FGFR1 inhibitor AZD4547 in combination with cisplatin, the cytoreductive effect was enhanced compared with cisplatin alone, indicating that FGFR1 inhibition could improve treatment efficacy.

Survivin, a protein that inhibits apoptosis, is highly expressed in LGACC, and research suggests that targeting survivin could promote cancer cell death [55]. Arsenic trioxide (As_2_O_3_), a broad-spectrum anti-tumor drug, can inhibit the high expression of survivin in LGACC, leading to increased apoptosis [55]. The extract of Ginkgo biloba (EGB) inhibits survivin gene expression in ACC-2 cells, induces apoptosis, and suppresses tumor cell proliferation, suggesting its potential as a therapeutic target [56]. Mulay et al. [57] conducted an in-depth study on survivin, scoring nuclear survivin expression based on expression intensity and percentage. They found that patients with high nuclear survivin scores (NS-SCORE) had shorter progression-free survival suggesting that survivin may serve as a prognosis marker for LGACC.

High-grade transformation (HGT) is a phenomenon in LGACC where tumor cells lose differentiation, increasing tumor aggression [58]. Studies have indicated that HGT-LGACC exhibits high proliferation rates, Ki-67 positivity, and increased p53 staining [59]. Additionally, HGT-LGACC demonstrates significantly lower expression of miR-29a-3p, a microRNA that targets the 3 'untranslated region (UTR) of the Quaking gene, thereby inhibiting proliferation, migration, and epithelial-mesenchymal transformation of LGACC cells [60]. AKT2 is a direct target gene of miR-29a-3p [61]. The expression levels of AKT2 and its phosphorylated form (p-AKT2) are elevated in hyperproliferative LGACC tissues, suggesting that AKT2 signaling may be activated in HGT-LGACC, which in turn indicates that inhibiting the expression of the AKT2 gene may be a therapeutic target for HGT-LGACC.

New potential targets in LGACC specimens reported by various researchers have been listed in Table 1.

**Table 1:** LGACC related genes

LGACC suppressor gene	1. bcl-2, bas [62]
LGACC promoter gene	2. surviving [55] 3. p53 [62] 4. Leptin [63] 5. GSTpi [64] 6. KRAS, MET, NRAS [65] 7. p63/CD117 [66] 8. VEGF [67] 9. MVD, MMP-2, MMP-9 [68] 10. PI3K-Akt, IL-17 [69] 11. glycine, serine, and threonine [70] 12. GLS1, ASCT2, MCT4 [71], 13. SHMT1, PSPH [71]

GSTpi, Glutathione S-transferase pi; KRAS, Kirsten Rat Sarcoma Viral Oncogene Homolog; MET, MET Proto-Oncogene; NRAS, NRAS Proto-Oncogene; VEGF, Vascular Endothelial Growth Factor; MVD, Mevalonate Diphosphate Decarboxylase; MMP-2, Matrix Metallopeptidase-2; MMP-9, Matrix Metallopeptidase-9; PI3K, Phosphatidylinositol-3-kinase; IL-17, Interleukin-17; GLS1, Glutaminase-1; ASCT2, Alanine-serine-cysteine transporter-2; MCT4, Monocarboxylate transporter-4; SHMT1, Serine Hydroxymethyltransferase-1; PSPH, Phosphoserine Phosphatase

Phase II clinical trials on targeted therapies for ACC, including FGFR, VEGFR, and PDGFR inhibitors, have yielded limited success. Which is showed in Table 2. These results indicate that targeted therapies showed modest objective response rate and promising median progression-free survival time range from 5.7 m to 17.7 m. However, further research is needed to improve their effectiveness.

**Table 2:** Phase II clinical trials on targeted therapies for ACC

Medicine	Targeted inhibitors	Objective response rate	Median progression-free survival (months)
Dovitinib [68]	FGFR, VEGFR, PDGFR	6%	8.2
Lenvatinib [69]	VEGFR, FGFR, PDGFR	16%	17.5
Axitinib [70]	VEGFR, PDGFR, KIT	9%	5.7

## Conclusion

Currently, the standard treatment for LGACC includes surgery followed by adjuvant radiotherapy. Studies have explored various surgical techniques and NIAC for difficult-to-resect tumors, though the long-term efficacy and toxicity of these approaches require further evaluation. Emerging radiotherapy techniques, such as proton and carbon-ion therapy, are promising but not yet widely adopted due to their high cost and limited availability. On the molecular front, targeted therapies hold potential for treating relapsed or metastatic LGACC, but challenges remain in their clinical application and requires further research.

In summary, we recommend eye-sparing surgery as the primary surgical approach for patients with T1–T2 stage tumors, supplemented with radiotherapy and chemotherapy. For patients with T3–T4 stage tumors, we suggest orbital exenteration as the main surgical option, also supported by radiotherapy and chemotherapy. If medical facilities permit, NIAC can be considered as an adjunctive treatment. For patients with advanced-stage tumors, targeted molecular therapies may be explored as potential treatment options.

## Data Availability

No datasets were generated or analysed during the current study.
